# Assistierter Suizid: Begutachtung und Beurteilung der Fähigkeit zur freiverantwortlichen Entscheidung

**DOI:** 10.1007/s00103-026-04197-8

**Published:** 2026-02-10

**Authors:** Norbert Nedopil, Peter Brieger

**Affiliations:** 1 Abteilung für Forensische Psychiatrie, Klinik und Poliklinik für Psychiatrie und Psychotherapie, LMU Klinikum, Universität München, Nussbaumstr. 7, 80336 München, Deutschland; 2 kbo-Isar-Amper-Klinikum Region München, Haar bei München, Deutschland

**Keywords:** Rechtliche Gepflogenheiten, Recht auf Sterben, Rechtsprechung, Psychische Gesundheit, Forensische Begutachtung, Legal thinking, Right to die, Legislation & jurisprudence, Mental health, Forensic medicine

## Abstract

Der Beitrag befasst sich mit der Frage, wie die Fähigkeit zur freiverantwortlichen Entscheidung beim assistierten Suizid begutachtet und beurteilt werden kann. Ausgehend von medizinethischen und rechtlichen Grundlagen sowie internationalen Vergleichen wird dargelegt, dass die Entscheidung zum Suizid besondere Anforderungen an Autonomie, Willensbildung und innere Festigkeit stellt. Der Begriff der „Freiverantwortlichkeit“ wird in Abgrenzung zu Geschäftsfähigkeit und Einwilligungsfähigkeit analysiert und seine Nähe zu diesen Konzepten eingeordnet.

Vorgeschlagen wird eine Operationalisierung, die Verständnis, autonome Wertung, nachhaltige Entscheidung und Fähigkeit zum selbsttätigen Handeln einschließt. Zudem werden praktische Herausforderungen für die Begutachtung diskutiert, insbesondere hinsichtlich der Rolle von Psychiater:innen, der Trennung von Funktionen und der Sicherstellung ergebnisoffener Verfahren.

Der Artikel plädiert für eine enge Definition der Freiverantwortlichkeit und eine standardisierte Begutachtungspraxis in Anlehnung an gängige und erprobte Begutachtungskonzepte der forensischen Psychiatrie, um sowohl den Schutz vulnerabler Personen als auch das Grundrecht auf Selbstbestimmung zu gewährleisten.

## Abstract (en)

This article addresses the question of how the capacity to make a freely responsible decision in assisted suicide can be assessed and evaluated. Based on medical ethics and legal principles as well as international comparisons, it is argued that the decision to commit suicide places special demands on autonomy, decision-making and inner strength. The concept of “free responsibility” is analysed in contrast to legal capacity and capacity to consent and is located in its proximity to these concepts.

An operationalisation is proposed that includes understanding, autonomous evaluation, sustainable decision-making and the ability to act independently. In addition, practical challenges for assessment are discussed, particularly with regard to the role of psychiatrists, the separation of functions and ensuring open-ended procedures.

The article advocates a narrow definition of free responsibility and a standardised assessment practice based on established and proven concepts of assessment in forensic psychiatry in order to guarantee both the protection of vulnerable persons and the fundamental right to self-determination.

## Vorbemerkungen

Das Urteil des Bundesverfassungsgerichts (BVerfG) vom 26.02.2020 zum „Verbot der geschäftsmäßigen Förderung der Selbsttötung“ stellte fest: „Das allgemeine Persönlichkeitsrecht (Art. 2 Abs. 1 i. V. m. Art. 1 Abs. 1 GG [Grundgesetz]) umfasst als Ausdruck persönlicher Autonomie ein Recht auf selbstbestimmtes Sterben“ [1]. Die Frage, wie die Fähigkeit zur freiverantwortlichen Entscheidung beim assistierten Suizid begutachtet und beurteilt werden kann, ist vielschichtig und noch keinesfalls abschließend beantwortet. Dieser Text versucht, entsprechende Antworten zu geben und basiert auf Denkansätzen des Erstautors, die bereits 2022 [2] publiziert wurden. Bislang zu dem Thema veröffentlichte Ausführungen werden hierbei berücksichtigt (z. B. [3, 4]).

Das Thema wird zuerst aus dem Blickwinkel der forensischen Psychiatrie betrachtet: Medizinisch psychiatrische Erkenntnisse und empirisches Wissen über psychische Störungen werden für nichtmedizinische Entscheidungsträger:innen übersetzt. Es geht um die Übertragung medizinisch psychiatrischen Fachwissens in rechtliche und andere normative Vorgaben und Gepflogenheiten und um die Aufarbeitung empirischer Forschung und wissenschaftlicher (im Sinne von naturwissenschaftlicher und erfahrungswissenschaftlicher) Erkenntnisse für die Umsetzung in einen normativen, juristischen und ethischen Kontext [5]. Doch ist dieses Konzept bei der Frage der Entscheidung zum Suizid und dem praktischen Vorgehen zur Selbsttötung gemäß dem Willen der Betroffenen nicht wirklich anwendbar. Das Erleben und die Konsequenz eines Suizids aus der subjektiven Sicht von Betroffenen ist erfahrungswissenschaftlich nicht zu erforschen. Es gibt keine empirischen Untersuchungen darüber, ob der Suizid die richtige Entscheidung war, zur Zufriedenheit beigetragen hat oder rückblickend für falsch erachtet wurde. Dieses Unwissen müsste eigentlich bei der Aufklärung über den Suizid und die Alternativen zum Suizid thematisiert werden.

Allerdings können Menschen, deren Suizidabsichten gescheitert sind, nach ihrer rückblickenden Einschätzung gefragt werden, ob sie dieses Scheitern bedauern oder damit zufrieden sind. Aus Katamnesen ist bekannt, dass die übergroße Mehrzahl der Menschen, die einen Suizidversuch überlebt, darüber auch Jahre später froh ist [6]. Dabei greift es zu kurz, Suizidabsichten ausschließlich auf das Vorliegen einer psychischen Erkrankung zurückzuführen. Unstrittig ist, dass diese das Risiko von Suizid erhöhen – es gibt aber durchaus auch Suizide, die ohne Vorliegen einer psychischen Erkrankung durchgeführt werden [7]. Dies stimmt mit der Aussage des Deutschen Ethikrats überein: „‚Suizidalität‘ steht für ein breites Spektrum an personalen, sozialen und gesellschaftlichen Phänomenen. Neben individuellen Faktoren nehmen auch die soziale Nah- und die gesellschaftliche Umwelt Einfluss darauf, ob und wie Suizidgedanken entstehen, verstärkt oder abgeschwächt werden“ [8, S. 10, Nr. 4].

Die Überlegungen in diesem Beitrag basieren auf folgenden wesentlichen Faktoren:

1. auf der medizinethischen Grundlage, die den heutigen überwiegenden Konsens in unserer Gesellschaft widerspiegelt [9]: Die Autonomie der Patient:innen steht an oberster Stelle der Hierarchie der ethischen Entscheidungsgründe für medizinisches Handeln;
2. auf dem Vergleich mit anderen Ländern, in denen in den letzten 3 Jahrzehnten gesetzgeberische und gerichtliche Entscheidungen unterschiedliche Formen der Beihilfe zum Suizid als rechtlich zulässig angesehen haben;
3. auf den subjektiven klinischen Erfahrungen, welche die Autoren mit Suizident:innen, Menschen mit Suizidgedanken, Menschen, die den Freitod wünschten, und mit der psychiatrischen Betreuung von Menschen, deren Angehörige durch einen unnatürlichen Tod aus dem Leben geschieden sind, gewonnen haben;
4. auf den Überlegungen, die gegenwärtig in Deutschland bezüglich des selbstgewählten und des assistierten Sterbens angestellt werden, insbesondere auf dem Urteil des BVerfG vom Februar 2020 [1], den Stellungnahmen des interdisziplinären Arbeitskreises der Deutschen Akademie der Wissenschaften Leopoldina [10] und des Ethikrates [8];
5. auf Überlegungen, die der Erstautor zusammen mit einer Arbeitsgruppe von Psychiater:innen, Psycholog:innen und Jurist:innen zur Überprüfung der Einwilligungsfähigkeit zur Behandlung bei Menschen mit psychischen Störungen, inklusive Demenz und mentaler Einschränkungen, um die Jahrtausendwende publiziert hat [11].

Aufbauend auf diesen Punkten wird im Beitrag diskutiert, wie die Fähigkeit zur freiverantwortlichen Entscheidung beim assistierten Suizid begutachtet und beurteilt werden kann. Die Begriffe freier Wille und Einwilligungsfähigkeit sowie die Besonderheiten von Freiverantwortlichkeit werden erläutert. Überlegungen zur Operationalisierung der Beurteilung der Freiverantwortlichkeit und die Umsetzung in der Praxis sowie ihre Herausforderungen und offene Fragen werden beschrieben.

## Autonomie und Willensentscheidungen

In der Regel wird die Autonomie eines Menschen als Grundlage für Entscheidungen bezüglich des eigenen Wohlergehens und der Gewährung oder Versagung von Eingriffen in den Vordergrund gestellt. Diese Autonomie wird im deutschen Rechtssystem durch den freien Willen des Menschen begründet. Der Begriff des „freien Willens“ wird im Urteil des BVerfG vielfach herangezogen, um die Freiverantwortlichkeit zum „assistierten Suizid“ (in der Schweiz: „assistiertes Sterben“, Deutsche Gesellschaft für Psychiatrie und Psychotherapie, Psychosomatik und Nervenheilkunde e. V. (DGPPN): „Suizidassistenz“) zu begründen. Allerdings hat eine Reihe von Begriffen, die den Willen des Menschen oder seine Autonomie beschreiben, im forensischen oder medizinethischen Gebrauch einen mehr oder weniger großen Überlappungsbereich.

Am besten definiert ist der *freie Wille*. Er wird im Gesetz (§ 104 Bürgerliches Gesetzbuch, BGB) in Zusammenhang mit der Geschäftsunfähigkeit aufgeführt, die auf einem den „freien Willen“ ausschließenden Zustand krankhafter Störung der Geistestätigkeit beruht. Gleichzeitig wird festgelegt, dass die Voraussetzungen für volle Geschäftsfähigkeit erst ab dem Alter von 18 Jahren vorliegen. Aus psychiatrisch-psychologischer Sicht ist es für eine freie Willensentscheidung erforderlich, dass der Mensch seine Bedürfnisse und Intentionen sowie die an ihn gestellten Anforderungen integrieren, seine eigenen Intentionen mit den Denk- und Handlungsalternativen bewusst abwägen und seine Willensentscheidung kommunizieren und ggf. relativieren kann.

Demgegenüber ist die *Einwilligungsfähigkeit*, die sich meist auf die Einwilligung in einen medizinischen Eingriff bezieht, nicht mit einer Altersgrenze verbunden, sondern von der Verstandesreife und davon abhängig, ob die betreffende Person Art, Bedeutung und Tragweite (Risiken) der (ärztlichen) Maßnahme und auch ihres Unterlassens erfassen und gegeneinander abwägen kann. Je komplexer und je schwerwiegender die Maßnahme ist und je nachhaltiger die Folgen sein können, desto höher sind auch die Anforderungen an die Einwilligungsfähigkeit. Im Gegensatz zu Entscheidungen, die Geschäftsfähigkeit erfordern, sind Entscheidungen, die von der Einwilligungsfähigkeit abhängen, widerrufbar.

Eine deutlich niedrigere Schwelle wird mit dem „*natürlichen Willen*“ verbunden. Er bezieht sich auf die tatsächlich vorhandenen Absichten, Wünsche, Wertungen und Handlungsintentionen eines Menschen, auch wenn er minderjährig ist oder sich in einem die freie Willensbestimmung ausschließenden Zustand krankhafter Störung der Geistestätigkeit befindet, d. h. für jeden Menschen ab der Geburt.

## Freiverantwortlichkeit

Willensäußerungen erfordern für verschiedene Entscheidungen in verschiedenen sozialen Situationen unterschiedliche kognitive und mentale Kompetenzen.

Die Willenserklärung zum Sterben unterscheidet sich von anderen Formen der Willensäußerungen und ihren Voraussetzungen in folgenden Aspekten:

- Die Entscheidung zum Suizid ist – ab dem Beginn ihrer Umsetzung – nicht widerrufbar und führt zwangsläufig zum Tod.
- Die Handlungskompetenz für den Suizid liegt beim Menschen mit Suizidabsichten (und nicht bei demjenigen, der Assistenz gewährt – der Bundesgerichtshof hat hier in einer aus anderen Gründen kritisch diskutierten Entscheidung den Begriff „Tatherrschaft“ benutzt [12]).
- Es gibt nur sehr begrenzt empirische und außerdem keine subjektiven Erfahrungswerte für das Ergebnis der Entscheidung.
- Die Entscheidung zum Suizid betrifft in besonderem Maße weit mehr das nahe soziale Umfeld als Willenserklärungen, die eine Einwilligungsfähigkeit voraussetzen.
- Die Willensentscheidung zum Suizid bedarf der „Festigkeit“, d. h. eines dauerhaften Wunsches, sterben zu wollen, und der Überzeugung, damit subjektiv das Richtige zu tun.

Der Ethikrat formuliert hierzu: „Selbstbestimmung hat kognitive, emotionale wie auch voluntative Komponenten und ist darüber hinaus an bestimmte soziale Bedingungen gebunden. Die für die zutreffende Entscheidung relevanten Umstände müssen als Teil der für das eigene Erleben maßgeblichen Welt begriffen, bewertet und verarbeitet werden. Ohne diesen Bezug auf die eigene, stets auch emotional getönte und sozial bestimmte Lebenswelt hat theoretische Erkenntnis keine praktische Bedeutung und kann daher nicht handlungswirksam werden“ [8, S. 14, Nr. 16].

Aufgrund dieser Unterschiede zu anderen Willensentscheidungen ist es sinnvoll, einen eigenen Begriff einzuführen, der als Voraussetzung für die Umsetzung des Suizids festgestellt werden muss. Vom BVerfG und von verschiedenen Autor:innen wurde hierfür der Begriff „Freiverantwortlichkeit“ gewählt [3, 10].

Die Einordnung dieses Begriffs in die Hierarchie der Anforderungen, die mit Willensentscheidungen verknüpft sind, ist juristisch noch nicht geklärt. Prima vista könnte eine Nähe zur Testierfähigkeit als Fähigkeit einer Person, ein gültiges Testament zu errichten, zu ändern oder aufzuheben (§ 2229 Abs. 4 BGB), bestehen, da es sich in beiden Fällen um letztwillige Verfügungen handelt. Aus medizinischer Sicht und dem Urteil des BVerfG ist jedoch eher eine Nähe zur Einwilligungsfähigkeit abzulesen.

Zur Klärung des Konzeptes „Freiverantwortlichkeit“ ist es sinnvoll, sich einerseits auf bekannte Vorgehensweisen bei vergleichbaren Fragestellungen zu berufen, andererseits die Inhalte der für die „Freiverantwortlichkeit“ geforderten Kompetenz operationalisiert abzubilden.

Wie schwierig die Lösung im konkreten Fall tatsächlich ist, soll anhand des psychiatrisch-psychologischen Wissens über Willensentscheidungen veranschaulicht werden. Die entwicklungspsychologischen Erkenntnisse über die Grundlage subjektiver Entscheidungen besagen, dass dabei insbesondere die biologische Entwicklung und Reife, das akquirierte Wissen, die bisher im Leben gewonnenen Kenntnisse und die individuellen Erfahrungen von entscheidender Bedeutung sind. Das Wissen und die Erfahrungen kommen durch Versuch und Irrtum und dem daraus resultierenden Verhalten (der Evaluierung), durch Nachahmung und durch pädagogischen Einfluss zustande [13]. Bezüglich des Todes fehlt eine Reihe der Grundlagen, die üblicherweise für Entscheidungen genutzt werden. Es scheint aus diesem Grund sinnvoll, die Definition der Freiverantwortlichkeit zum Suizid eng zu fassen, solange es keine erfahrungswissenschaftlichen Grundlagen für die Willensentscheidung zum Sterben gibt.

## Überlegungen zur Operationalisierung der Beurteilung der Freiverantwortlichkeit

### Analogien bei der Einwilligungsfähigkeit

Die Freiverantwortlichkeit hat in mehreren Aspekten die größte Nähe zu dem bereits seit Langem in der rechtlichen und medizinethischen Praxis gebräuchlichen Begriff der Einwilligungsfähigkeit (siehe auch: BVerfG 26.02.2020–2 BvR 2347/15 Rn. 242), wobei zu Recht immer wieder darauf hingewiesen wurde, dass die beiden Begriffe nicht gleichgesetzt werden dürfen (vgl. [3, 4]). Es bietet sich deshalb zunächst an, die Überlegungen zur Operationalisierung der Einwilligungsfähigkeit zu betrachten und zu überlegen, welche Aspekte sich auf die Freiverantwortlichkeit übertragen lassen [2, 14].

In Anlehnung an Amelung [15, 16] haben Nedopil et al. [11] für die Voraussetzungen der Einwilligungsfähigkeit Folgendes ausgeführt:

1. „Einwilligung bedeutet die Zustimmung zu einem persönlichen Opfer: Einwilligende opfern aus juristischer Sicht ein Rechtsgut und stimmen einer möglichen Beschädigung ihres Körpers zu. Dieses Opfer wird erbracht, um einem Nachteil zu entgehen oder einen Vorteil zu erhalten. Einwilligende müssen also ein subjektives Wertesystem besitzen, anhand dessen sie solche Entscheidungen vornehmen. Sie müssen somit die Fähigkeit zur autonomen Wertung besitzen.
2. Einwilligung bedeutet auch eine prognostische Entscheidung. Einwilligende müssen die Frage beantworten, welcher Eingriff ihnen in der Zukunft Vorteile bringen oder Nachteile verhindern wird. Sie müssen somit entweder über Informationen verfügen, die derartige prognostische Entscheidungen ermöglichen, oder sie müssen der Aufklärung über Tatsachen, die für ihre Entscheidung erforderlich sind, folgen können. Darüber hinaus müssen sie eine Vorstellung über Kausalzusammenhänge entwickeln können.
3. Letztendlich müssen Einwilligende nicht nur Alternativen erkennen können und einen subjektiven Wertmaßstab für die darin enthaltene Konfliktlösungsstrategie besitzen, sondern auch jene Alternative wählen können, von der sie sich den meisten Nutzen versprechen.“

Bei psychischen Störungen kann jede der 3 aufgeführten Voraussetzungen beeinträchtigt sein.

Vergleichbare Voraussetzungen lassen sich auch der internationalen Literatur entnehmen: Als Grundlagen für Einwilligungsfähigkeit gelten die Fähigkeiten zum Verstehen, zum Bewerten, zur rationalen Entscheidung (was wiederum die Fähigkeit zum folgerichtigen Denken, zum vergleichenden Beurteilen und zum Abwägen von Wahrscheinlichkeiten umfasst) und zur Kommunikation und Begründung einer Wahl [17]. Dabei liegen – basierend auf dem Modell von Grisso und Appelbaum [18] – grundsätzlich Konzepte zur Bestimmung der Einsichtsfähigkeit vor, die auch psychometrische Instrumente umfassen und deren Anwendung inzwischen auch für den Bereich des assistierten Suizids diskutiert werden [19]. Der Medizinethiker und Neurologe *Jox *hat ähnlich wie *Saks und Jeste *folgende 5 Voraussetzungen für die Annahme von Einwilligungsfähigkeit aufgeführt [20]:

- Verständnis: Der Betreffende muss den Sachverhalt, die Situation und die daraus abzuleitenden Konsequenzen verstehen.
- Anwendung: Er muss die Konsequenzen auf seine eigene spezifische Situation anwenden können.
- Abwägung: Er muss in der Lage sein, das Für und Wider einer Situation abzuwägen.
- Entschließung: Er muss aufgrund der Abwägung einen Entschluss fassen können.
- Kommunikation: Er muss diesen Entschluss kommunizieren können.

Einer derart positiven Zuschreibung der Einwilligungsfähigkeit stehen einige praktische Probleme entgegen. So erfordert die positive Zuschreibung die Feststellung der Einwilligungsfähigkeit in jedem Einzelfall, es müssten also alle 5 von *Saks und Jeste *[17] oder *Jox *[20] aufgezählten Kriterien auf ihr Vorhandensein geprüft werden. Bei einem solchen Vorgehen würde Einwilligungsfähigkeit möglicherweise auch bei einem Menschen verneint, der bewusst und willentlich Unsinniges intendiert. Es wird nämlich nicht gefragt, ob eine Krankheit oder Störung ausschlaggebend ist für die jeweilige Willensäußerung oder nur eine Laune, die auch gesunde Menschen haben können.

In den juristischen Gepflogenheiten wurden seit Langem für dieses Problem Lösungen gefunden, die aus Sicht der Autoren pragmatischer und vermutlich auch rechtlich sicherer sind: So hat es sich bewährt, das, was üblich oder normal ist, als gegeben hinzunehmen und als „Norm“ zu akzeptieren. Das Gesetz und die Rechtsanwender gehen somit davon aus, dass der (erwachsene) Mensch geschäfts-, testier-, ehe- oder schuldfähig ist, ohne die jeweiligen Begriffe zu definieren oder den Menschen grundsätzlich und in jedem Fall bezüglich dieser Fähigkeiten zu untersuchen. Eine Überprüfung ist erst dann erforderlich, wenn Zweifel an diesen Fähigkeiten bestehen. Definiert wird deshalb die Ausnahme von der Regel, die Geschäfts‑, Testier‑, Ehe- oder Schuld*un*fähigkeit. Diese Ausnahme gilt es zu beweisen. Wenn dieser Beweis nicht gelingt, ist vom Norm(al)zustand auszugehen.

Ein solches Denkmuster entspricht auch den klassischen ärztlichen Überlegungen, bei denen die Pathologie im Vordergrund steht und ärztliches Handeln sich primär an dieser und weitaus seltener an der unbeeinträchtigten physiologischen und psychologischen Funktionsfähigkeit orientiert. Ohne diese Hintergründe zu erwähnen, gehen auch Marckmann und Pollmächer [21] von einem solchen Konzept aus: Sie schreiben, dass eine akute psychische Krise oder Suizidalität als Symptom einer Störung Suizidassistenz ausschließen, weil der Wunsch zu sterben nicht einer autonomen (prämorbiden) Willensbildung entspricht. In dieselbe Denkrichtung geht auch der Hinweis auf die besondere Vulnerabilität von und die spezielle Gefahr bei Menschen mit psychischen Erkrankungen. Bei ihnen besteht eine ausgeprägte Wechselwirkung zwischen der psychischen Störung und der Suizidalität, die freie Willensbildung ist häufig beeinträchtigt und ihre Entscheidungen sind seltener gefestigt und stabil. Zudem ist es schwierig, „unerträgliches Leid“ objektiv zu erfassen und eine Unbehandelbarkeit des Leids zuverlässig festzustellen.

Darüber hinaus ist für jede Feststellung von rechtlich relevanten Einbußen durch psychische Störungen ein 2‑stufiges Vorgehen vorgesehen. Danach ist zunächst eine Krankheit oder Störung festzustellen, die einem juristischen Krankheitsbegriff zugeordnet werden muss (1. Stufe: Definition des Eingangsmerkmals). Erst wenn aufgrund dieser Störung eine für die Fragestellung relevante Funktionseinbuße vorliegt (2. Stufe), kann von der betreffenden Unfähigkeit ausgegangen werden. Auch dieses Vorgehen legt eine Orientierung an der Störung und deren Auswirkungen nahe und nicht eine positive Bestimmung der Kompetenzen für rechtsrelevantes Handeln.

### Besonderheiten der Freiverantwortlichkeit

Versucht man aus diesen Überlegungen die prinzipiellen Voraussetzungen für die Freiverantwortlichkeit abzuleiten, lassen sich hierfür folgende Fähigkeiten und Einstellungen feststellen:

- die Fähigkeit zum Erkennen von Tatsachen und Kausalverläufen und zum Ableiten von prognostischen Entscheidungen aus dieser Erkenntnis für sich selbst und andere. Der oder die Betreffende muss in der Lage sein, die Auswirkungen seines/ihres Handelns für sich und andere zu erkennen (Verständnis und Fähigkeit zum Perspektivenwechsel);
- die Fähigkeit zum eigenständigen Abwägen zwischen dem Suizid und den Überlebensalternativen und zur Begründung dieser Abwägung (autonome Wertung);
- die Fähigkeit zur Entscheidung zwischen Alternativen aufgrund eines subjektiven Wertmaßstabs durch eigene Erfahrungen (individuelle, persönliche Wertung und Konfliktlösung, Verarbeitung);
- Nachhaltigkeit und Dauerhaftigkeit der Entscheidung (innere Festigkeit);
- Fähigkeit und Bereitschaft zu selbsttätigem Handeln (autonomes Handeln).

Dann besteht bei jenem keine Freiverantwortlichkeit, dem es an innerer Festigkeit bezüglich des Sterbewunsches fehlt. Darüber hinaus ist auch derjenige unfähig zur Freiverantwortlichkeit, der wegen Minderjährigkeit, Krankheit oder dauerhafter kognitiver Beeinträchtigung (1. Stufe) unfähig ist,

- den für die Entscheidung relevanten Sachverhalt zu verstehen (Verständnis);
- die Entscheidung im Hinblick auf die eigene gegenwärtige Situation und die sich daraus ergebenden Folgen und Risiken für sich selbst und andere zu verarbeiten (Verarbeitung);
- zu erfassen, welchen Wert die betroffenen Interessen für sie/ihn haben (wichtig ist die Bezugnahme auf die – nicht durch Krankheit verzerrte – Werthaltung des Betroffenen; Bewertung);
- den eigenen Willen auf der Grundlage von Verständnis, Verarbeitung und Bewertung der Situation zu bestimmen (Bestimmbarkeit des Willens; 2. Stufe).

Die Fähigkeit zum Erkennen von Tatsachen und Kausalverläufen (Verständnis) kann z. B. bei wahnkranken Menschen durch falsche Beziehungssetzungen beeinträchtigt sein. Gestört sein kann zudem die Fähigkeit zur autonomen Wertung z. B. bei Personen mit Depression etwa durch nihilistische Gedankeneinengung und die Fähigkeit zur Konfliktlösung aufgrund einer persönlichen Wertung z. B. bei Demenz.

Wenngleich die (fehlende) Festigkeit des Willensentschlusses und die (Un‑)Fähigkeit zu selbstständigem Handeln auch bei Betroffenen ohne psychische Störungen geprüft werden müssen, gibt es jedoch psychische Störungen, bei denen Beeinträchtigungen in diesem Bereich vorkommen. Die Fähigkeit zu innerer Festigkeit kann beispielsweise bei emotionaler Instabilität, die Fähigkeit zur autonomen Tathandlung bei psychotischer Ambivalenz gestört sein.

## Überlegungen für die Praxis

Wie eine Begutachtung zur Freiverantwortlichkeit zu gestalten ist, ist aus Sicht der Autoren noch nicht umfassend geklärt. Die formaljuristischen Vorgaben und ihre Umsetzungen wurden in diesem Beitrag und von anderen [4] dargestellt. Nach formaler Prüfung (z. B. Ausschluss von Minderjährigkeit), Aufklärung, Beratung über denkbare Alternativen und Hilfsangebote sowie einer gewissen Reflexionsphase erfolgt in einem ersten Schritt der Ausschluss psychischer Krankheit oder kognitiver Beeinträchtigung (hierzu bedarf es der psychiatrischen Kompetenz). Bei psychischer Krankheit oder kognitiver Beeinträchtigung folgt im zweiten Schritt die Prüfung, ob dadurch Verständnis, autonome Wertung oder die Fähigkeit zur Konfliktlösung bei konkurrierenden Bestrebungen beeinträchtigt sind oder fehlen. Unabhängig von der Feststellung im ersten Schritt, das heißt, auch wenn Minderjährigkeit, (psychische) Krankheit oder kognitive Beeinträchtigung nicht vorliegen, ist die Prüfung, ob innere Festigkeit und Bereitschaft zur eigentätigen Handlung fehlen, erforderlich, da dann grundsätzlich Freiverantwortlichkeit nicht angenommen werden kann. Zudem ist auszuschließen, dass die betreffende Person äußeren Einflüssen, Nachahmungsbedürfnissen oder einem Druck unterliegt, dem er oder sie nicht widerstehen kann – auch wenn diese Frage nicht notwendigerweise eine gutachterliche ist.

Zusammenfassend sollten die in der Infobox dargestellten Fragen abgeklärt werden.

Wenig beleuchtet scheint die konkrete Ausführung der Begutachtung.

Welche Beziehung entsteht zwischen Gutachter:innen und Betroffenen, wenn der Auftrag der Betroffenen an die Gutachter:innen ist, den Tod zu ermöglichen? Welche Auswirkungen hat das auf die Interaktion – seien sie emotional-therapeutisch oder finanziell (Vorauskasse)? Wie verabschiedet sich der/die Gutachter:in von einem Menschen, dessen Tod durch das Gutachten ermöglicht wird?

Einige Erkenntnisse dazu (z. B. auch zu den Geldflüssen) liegen durch die Arbeitsgruppe um Sabine Gleich vor, die Untersuchungen zur momentanen Rechts- und Begutachtungspraxis bei assistieren Suiziden in München durchgeführt hat [22]. Es wird berichtet [23], dass aktuell „keine adäquaten Schutzkonzepte für die vulnerable Klientel“ umgesetzt seien. Die dokumentierten Gespräche und Begutachtungen erschienen häufig nicht ergebnisoffen, was sich auch darin widerspiegelte, dass „Beratung bzw. Begutachtung, Assistenz und Leichenschau in fast der Hälfte der untersuchten Fälle in der Hand eines einzigen Arztes“ lagen. Suizidenten mit psychiatrischen Vorerkrankungen und gesetzlicher Betreuung wurden ganz überwiegend „nicht von Fachärzten für Psychiatrie begutachtet“.

Die Arbeitsgruppe fordert deswegen u. a. die obligate „Trennung von vorbehandelndem und aufklärendem Arzt, Gutachter, assistierendem Arzt sowie Leichenschauer“ [24]. Darüber hinaus wird „eine Überprüfung der Eignung der Gutachter“ als „wünschenswert“ eingeschätzt: „Jedenfalls bei Erkrankungen mit einem potenziellen Einfluss auf die Freiverantwortlichkeit wie Depressionen, kognitiven Einschränkungen und Demenz, bei Betreuung oder sonstigen Anhaltspunkten für mangelnde Freiverantwortlichkeit sollten aus Sicht der Autoren Fachärzte für Psychiatrie in die Beurteilung miteinbezogen werden. Entsprechende GA [Gutachten] sollten standardisiert abgefasst werden. Mindestanforderungen wie für Schuldfähigkeitsgutachten sollten festgelegt werden.“ Dabei wird darauf verwiesen, dass mehrere vorgelegte Gutachten entsprechende Voraussetzungen nicht erfüllten, sondern tatsächlich Atteste waren. Die Autoren sehen hier, wie die Arbeitsgruppe um Gleich, unbedingten Handlungsbedarf des Gesetzgebers oder hilfsweise der Gerichte.

## Fazit

Das Diktum des BVerfG, dass das „Recht auf selbstbestimmtes Sterben … die Freiheit ein[schließt], sich das Leben zu nehmen“, stellt die Begutachtung solcher Fragen vor Herausforderungen. Schutzkonzepte für vulnerable Personengruppen wie auch Konzepte der Suizidprävention sind neben den dargestellten Überlegungen unabdingbar.

### Infobox Fragen zur Beurteilung der Freiverantwortlichkeit

Hat der/die Betreffende die Aufklärung verstanden?

Wurden die Alternativen reflektiert und begründet zurückgewiesen?

Entspricht der Suizidwunsch einem überdauernden Wertgefüge?

Ist der/die Betreffende zu einem Perspektivenwechsel in der Lage?

Will der/die Betreffende die Handlungskompetenz delegieren?
